# Motor fMRI and cortical grey matter volume in adults born very preterm

**DOI:** 10.1016/j.dcn.2014.06.002

**Published:** 2014-06-17

**Authors:** E.J. Lawrence, S. Froudist-Walsh, R. Neilan, K.W. Nam, V. Giampietro, P. McGuire, R.M. Murray, C. Nosarti

**Affiliations:** a Department of Psychosis Studies, Institute of Psychiatry, King's Health Partners, King's College London, De Crespigny Park, London SE5 8AF, UK; b Department of Neuroimaging, Institute of Psychiatry, King's Health Partners, King's College London, De Crespigny Park, London SE5 8AF, UK; c Centre for the Developing Brain, King's Health Partners, King's College London, First Floor, South Wing, St Thomas’ Hospital, London SE1 7EH, UK

**Keywords:** Preterm, Adult, fMRI, Motor, Cognitive, Neurodevelopment

## Abstract

The primary aim of this study was to investigate the functional neuroanatomy of motor planning, initiation and execution in a cohort of young adults (mean age 20 years) who were born very preterm (VPT; <33 weeks of gestation), as these individuals are at increased risk of experiencing neuromotor difficulties compared to controls. A cued motor task was presented to 20 right-handed VPT individuals and 20 controls within a functional magnetic resonance imaging (fMRI) paradigm. Whole-brain grey matter volume was also quantified and associations with functional data were examined. Despite comparable task performance, fMRI results showed that the VPT group displayed greater brain activation compared to controls in a region comprising the right cerebellum and the lingual, parahippocampal and middle temporal gyri. The VPT group also displayed decreased grey matter volume in the right superior frontal/premotor cortex and left middle temporal gyri. Grey matter volume in the premotor and middle temporal clusters was significantly negatively correlated with BOLD activation in the cerebellum. Overall, these data suggest that preterm birth is associated with functional neuronal differences that persist into adulthood, which are likely to reflect neural reorganisation following early brain injury.

## Highlights

• Adults born very preterm (VPT) and controls performed a motor fMRI task.
• VPT adults activated the cerebellum and adjacent temporal lobe more than controls.
• Grey matter volume in the premotor cortex was smaller in the VPT group.
• Grey matter volume in premotor cortex explained 33% of activation in the cerebellum.
• Preterm birth is associated with functional neuroanatomical alterations in adulthood.

## 1 Introduction

Children and adolescents who were born before 33 weeks of gestation, or ‘very preterm’ (VPT) display differences on measures of neurocognitive function compared to controls, which have in part been associated with alterations in brain structure (Nosarti et al., 2008). Following these individuals into adulthood is essential to determine the degree and nature of alteration in their developmental trajectory, and to understand possible processes of neural plasticity following adverse perinatal events. Ultimately, these data are of importance in evaluating the need for neuroprotective remediation strategies.

Several studies suggest that VPT children underperform in the motor domain (Bracewell and Marlow, 2002, Spittle et al., 2011, Van Braeckel et al., 2010) with up to 30% of those aged 7–8 displaying motor impairment (Foulder-Hughes and Cooke, 2003) and delays in visuomotor development (Atkinson and Braddick, 2007). Motor deficits may also persist into young adulthood, with VPT individuals displaying increased motor confusion and sensory integration (Allin et al., 2006). Neurosensory disability in VPT children has been found to correlate with impaired cognitive functioning (Sherlock et al., 2005), suggesting that cognitive outcome may be confounded by neuromotor deficits. Furthermore, some studies have shown that if individuals with neuromotor deficits are excluded from analysis, differences in cognitive scores between children with and without preterm-birth related periventricular injury become attenuated (Costello et al., 1988, Wood et al., 2005).

These neuromotor deficits may be in part due to structural differences in the VPT brain (Atkinson and Braddick, 2007, Nosarti et al., 2008, Spittle et al., 2011). Both the thalamus and the caudate nucleus, key brain structures in the motor network (Lehericy et al., 2006, Sommer, 2003), are prone to damage in VPT neonates (Ball et al., 2012). Reductions in grey matter volume in the thalamus and caudate have been measured in VPT children and adolescents and have also been shown to be related to neuropsychological outcome including IQ, verbal fluency, language and executive function, visual attention and visuospatial integration (Abernethy et al., 2004, Gimenez et al., 2006, Nagasunder et al., 2011, Nosarti et al., 2008), in line with the idea that neurological and cognitive functions may share underlying brain circuitry.

However, data on the adult neuroanatomical correlates of motor function following preterm birth are limited. Functional magnetic resonance imaging (fMRI) may have the additional sensitivity required to detect subtle differences in VPT adults that behavioural data do not reveal. For instance, altered brain activation during attention allocation and motor response inhibition tasks has been observed in VPT adolescents (Nosarti et al., 2006) and adults (Lawrence et al., 2009), which may reflect a differential engagement of task-specific brain areas, as well as the use of alternative neuroanatomical substrates for task completion. These studies suggest differences in caudate nucleus activation at adolescence (Nosarti et al., 2006) and increased activation in brain regions that subserve less specialised cognitive processes in adulthood (Lawrence et al., 2009, Salvan et al., 2013).

The aim of the current study was to investigate the neural basis of motor planning, initiation and execution in young adults born VPT, as a means of assessing differences in the engagement of brain circuitry involved in neuromotor function, as well as studying potential compensatory/alternative neural processes in the VPT group. Grey matter volume was also quantified to investigate whether potential structural brain alterations would account for variations in blood-oxygen-level-dependent (BOLD) signal in the VPT cohort. We analysed relationships between BOLD signal and grey matter volume in areas where significant differences between VPT individuals and controls had been detected throughout the brain, as we hypothesised that structural differences in distant, connected parts of the neuromotor network could affect the function of areas displaying differential BOLD activation (Salvan et al., 2013). We hypothesised that adults born VPT would show altered BOLD signal compared to controls in task-specific fronto-parieto-cerebellar neural networks (Broome et al., 2010) and periventricular regions of the motor network, which are vulnerable to perinatal injury and developmental alterations in VPT cohorts i.e. caudate nucleus and thalamus.

## 2 Method

### 2.1 Participants

In 1983–1984, the Neonatal Unit at University College London Hospital (UCLH), admitted 252 infants born at <33 weeks gestation within 5 days of birth. These infants survived and were discharged. Of this group, all the infants who were born at ≤28 weeks of gestation were enrolled for follow-up, alongside a random sample of those born from 29 to 33 weeks gestation. Limited research resources prevented inclusion of the entire consecutive series. One hundred and forty-seven adolescents, or 40% of the entire sample, were selected for study (all those born at <28 weeks gestation (*n* = 78) and a random selection of 69 individuals born at 29–33 weeks gestation). One hundred and thirteen individuals were assessed in adolescence; 57 were born at ≤28 weeks and 56 from 29 to 33 weeks gestation. Participants who did not meet the exclusion criteria i.e. history of any of the following: cerebral palsy, periventricular leucomalacia, severe hearing impairment, visual impairment, motor impairment or grade 3/4 intraventricular haemorrhage (who represented 17% of our sample at a previous assessment (Nosarti et al., 2008)), were contacted and those who responded took part in a series of studies. For the present fMRI study 20 right-handed VPT individuals participated (12 born at ≤28 weeks gestation, and 8 born at 29–31 weeks gestation; 14 males; mean age 20.2 ± 0.66 years). This subsample did not differ from the larger cohort assessed in mid-adolescence (Nosarti et al., 2008) in terms of gestational age (*t*_(111)_ = 1.3; *p* = 0.19), birth weight (*t*_(111)_ = 0.69; *p* = 0.49), gender distribution ( $\chi_{\left(1\right)}^{2}=2.0;$*p* = 0.16) and neurological status as assessed by a clinical neurological examination at age 8 years, which classified results as normal (i.e., no detected impairment), equivocal (i.e., when signs but no definite abnormality were detected), and abnormal (i.e., when a definite abnormality was observed) ( $\chi_{\left(2\right)}^{2}=2.1;$*p* = 0.35). None of the study participants had a diagnosis of developmental co-ordination disorder.

Twenty right-handed controls were recruited from local advertisements in the press and universities (nine males; mean age 19.72 ± 1.8). The inclusion criteria were full-term birth defined as 37–42 completed weeks of gestation and English as a first language. The exclusion criteria included birth complications (e.g. low birth weight defined as <2500 g, endotracheal mechanical ventilation), gestation greater than 42 weeks, a history of psychiatric illness, severe hearing, visual or motor impairment.

Ethical approval for the study was obtained from the local ethics committee. Written informed consent for the assessment, including MRI, was also obtained from all participants.

### 2.2 MRI imaging parameters

Echo planar MR images showing BOLD contrast were obtained using a 1.5 Tesla GE Signa Neurovascular MR system (GE Medical Systems, Milwaukee, WI, USA). Radio frequency transmission and reception was accomplished using a quadrature head coil. To enable the mapping of the functional data into Talairach space, we also acquired a high-resolution inversion recovery EPI data-set with 3 mm thick near-axial slices and an in-plane resolution of 1.5 mm. The random motor movement task was studied using a gradient-echo sequence (TR 2000 ms, TE 40 ms, flip angle 90°). In each of the twelve 5.5 mm thick near axial slices with a 0.5 mm gap, 150 T2-weighted MR images depicting BOLD contrast were acquired parallel to the intercommissural (AC-PC) plane so as to include the whole brain. Three-dimensional T1-weighted gradient-echo sequences were also collected that allowed reconstruction in any plane of 124 1.5 mm slices (TR 35 ms, TE 5 ms, flip angle 35°, inplane resolution 0.86 × 086) to allow the measurement of brain structures of interest.

### 2.3 Motor paradigm

This study used an fMRI block design in a random movement generation task (Broome et al., 2010). During the active condition, participants were presented with the word ‘MOVE’ on a computer screen for 0.5 s, at the centre of 4 arrows pointing north, south, east and west away from the word. Participants had been instructed to move a joystick once, in a randomly chosen direction, as quickly as possible when they saw the word, in any one of the four directions each time. All participants were asked to use their right dominant hand. The control baseline condition involved visual presentation of the word ‘REST’. We explained to participants that when they saw this word they should not move the joystick. Fifteen stimuli were presented at 2 s intervals in blocks of 30 s in both conditions. Five blocks of each condition were presented, and the order was alternated. We recorded the reaction time for each trial.

### 2.4 Functional MRI analysis

#### 2.4.1 Individual and group mapping

The fMRI data were analysed with the XBAM v.3.4 software developed at the Institute of Psychiatry (c.f.www.kcl.ac.uk/iop/depts/neuroimaging/research/imaginganalysis/Software/XBAM.aspx). This software uses a non-parametric permutation-based strategy to minimise assumptions. Firstly, the images were corrected for subject motion and then they were smoothed using a Gaussian filter (FWHM 8.8 mm). BOLD response to the experimental paradigm were detected by fitting a linear model in which each aspect of the design was convolved separately with two gamma variate functions with peak responses at 4 s and 8 s post stimulus presentation. The weighted sum of these convolutions that best fit the time-series at each voxel was calculated using the constrained BOLD effect model (Friman et al., 2003). This allows for variability in the haemodynamic delay and decreases the possibility of obtaining mathematically, but not physiologically plausible results. Next, a goodness of fit statistic was computed. This consists of the ratio of the sum of squares of deviations from the mean image intensity due to the model (over the whole time series) to the sum of squares of deviations due to the residuals (SSQ). The data were permuted using a wavelet-based method (Bullmore et al., 2001) which enables the data-driven calculation of the null distribution of SSQ under the assumption of no experimentally-determined response. The percentage BOLD change was also calculated from the model fit at each voxel. This analysis was extended from voxel to cluster level. The observed and randomised SSQ data for each individual were then normalised into standard Talairach space and group maps were computed using group median as a statistic. Permutation testing and median statistics were employed to allow exact computation of *p*-values with minimal assumptions and the minimisation of outlier effects (Brammer et al., 1997). The final cluster maps were statistically thresholded to obtain <1 false positive cluster per map, with a cluster size threshold of 20 voxels applied.

Group comparisons were performed by fitting the data at each brain voxel (in which all subjects had non-zero data) using a linear model, which was fitted by minimising the sum of absolute deviations, rather than the sum of squares, in order to reduce outlier effects. The null distribution was obtained by permuting subjects’ data between the groups, which makes use of the null hypothesis of no effect of group membership on BOLD signal, and refitting the linear model. Group difference maps are then generated by comparing the real data difference maps with the appropriately thresholded map of the null distribution.

### 2.5 Structural MRI analysis

All images were processed using VBM8 toolbox (C. Gaser, structural Brain Imaging Group, Department of Psychiatry, University of Jena; http://dbm.neuro.uni-jena.de/vbm/) in Statistical Parametric Mapping SPM8 (Wellcome Department of Cognitive Neurology, Institute of Neurology, London, UK; http://www.fil.ion.ucl.ac.uk/spm/software/spm8/), running on Matlab Version 7.8.0.347 (R2009a) (MathWorks, Natick, USA). As participants in the current study did not experience any significant medical complications in the perinatal period (e.g., cerebral palsy, grade 3/4 intraventricular haemorrhage or periventricular leukomalacia) we used default SPM8 tissue probability maps. Images were bias-corrected, skull-stripped and segmented into grey matter, white matter and CSF before being normalised to the standard template using DARTEL. Images were modulated, correcting for the non-linear part of the native-to-standard space warp and smoothed with a 12 mm × 12 mm × 12 mm kernel. Independent sample *t*-tests were used to compare the groups. Family wise error (FWE) correction was applied on voxel level whole-brain analysis results. In the regions where significant grey matter differences were observed between the VPT and the control group, grey matter eigenvalues were extracted for each cluster in each scan using SPM's volume of interest (VOI) data extraction tool. One-sample Kolmogorov–Smirnov tests were also performed, and revealed a normal distribution of the data.

### 2.6 Demographic and behavioural data analysis

Intra-individual coefficient of variation (ICV) for response time was calculated as the standard deviation of the response time divided by the mean response time (de Zeeuw et al., 2008). Parametric tests were used where the data met assumptions i.e. interval data which does not seriously deviate from a normal distribution, homogeneity of variance between groups and independence of observations. Where the Levene's test for homogeneity of variance was significant, this is stated in the text and the values cited do not assume equal variance. Where the data seriously deviated from parametric assumptions, the non-parametric equivalent was used i.e. chi square for the categorical gender analysis and Mann–Whitney *U* test for number of non-responses which were heavily skewed due to a ceiling effect for most participants.

### 2.7 Associations between functional and structural MRI data and gestational age

Pearson correlations were used in regions of significant differences in the fMRI and structural MRI analysis to test for significant correlations between gestational age and BOLD activation and grey matter eigenvalues, respectively.

## 3 Results

### 3.1 Demographic and cognitive data

One control participant was excluded from the analyses due to technical problems during image acquisition. There were no differences between groups in age (*t*_(37)_ = −0.9; *p* = 0.39)^2^ or gender distribution, Full scale IQ, verbal IQ or performance IQ, as measured by the Wechsler Abbreviated Scale of Intelligence (Wechsler, 1999) – see Table 1.^3^

**Table 1:** Demographic, neonatal characteristics and behavioural data by group.

Mean and SD	VPT group (*n* = 20)	Control group (*n* = 19)	Mean difference	95% confidence interval of the difference	Test statistic	*p* value
Age at assessment in years	20.2 years ± 0.7	19.8 ± 1.8	0.38	−1.27 to 0.51	*t*_(37)_ = −0.9	*p* = 0.39
Gender (m:f)	14:6	8:11	–	–	$\chi_{\left(1\right)}^{2}=2.06$	*p* = 0.08
Gestation in weeks	28.1 ± 1.7	–	–	–		–
Birth weight in grams	1212 ± 259.3	–	–	–		–
Full scale IQ	96.7 ± 11.23	104.86 ± 14.01	8.16	−0.67 to 16.98	*t*_(32)_ = 1.9	*p* = 0.07
Verbal IQ	93.65 ± 13.01	101.86 ± 13.75	8.21	−1.24 to 17.66	*t*_(32)_ = 1.8	*p* = 0.09
Performance IQ	100.5 ± 10.73	106.71 ± 14.24	6.21	−2.5 to 14.93	*t*_(32)_ = 1.5	*p* = 0.16
Mean response time	0.48 ± 0.13	0.48 ± 0.13	0.01	−0.1 to 0.08	*t*_(34)_ = −0.15	*p* = 0.88
ICV of response time	0.63 ± 0.14	0.62 ± 0.08	0.01	−0.09 to 0.07	*t*_(34)_ = −0.24	*p* = 0.82
Non-responses (median, range)	0 (0–14)	0 (0–22)	–	–	*U* = 174	*p* = 0.57

### 3.2 On-line behavioural data

Due to technical problems with data collection, reaction times were not available for three participants. 71.8% of participants gave a response on every trial. There were no significant between group differences in number of non-responses, mean response times or response time ICV – see Table 1.

### 3.3 Functional MRI data

#### 3.3.1 Within-group activation

During the ‘Move’ condition when contrasted to ‘Rest’, control participants showed significant BOLD response in the left precentral gyrus, left insula, left medial frontal gyrus and the right inferior parietal lobule. The VPT group showed significant brain activation in a large cluster with local maxima in the left precentral gyrus (see Table 2).

**Table 2:** Cluster maxima for contrast for move > rest.

	Peak MNI co-ordinates			Cluster	No. of voxels
	*x*	*y*	*z*	*p* value	
Within group					
Control participants					
L precentral gyrus (BA4)	−34	−21	57	0.001	349
L insula (BA13)	−45	2	1	0.005	306
R inferior parietal lobe (BA40)	53	−33	45	0.006	167
L medial frontal gyrus (BA6)	−3	−10	50	0.007	97
VPT participants					
L precentral gyrus (BA4)	−34	−21	57	0.001	334
Between group					
VPT > control participants					
Cerebellum	21	−53	−23	0.003	65
Control > VPT participants					
Empty					

#### 3.3.2 Between-group comparisons

**Fig. 1:**
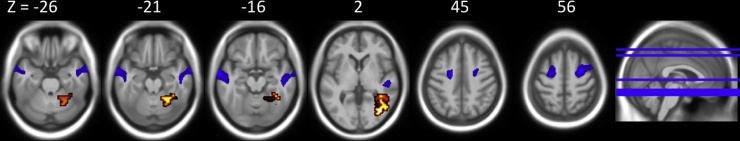
Foci of between-group activation for VPT ≥ control for move ≥ rest (hot) (*p* ≤ 0.05, corrected for multiple comparisons). Areas of decreased grey matter volume in the VPT group compared to controls are shown in blue (*p* ≤ 0.001, uncorrected for multiple comparisons). Structural and functional clusters are overlaid on the study-specific average template created from all participants’ T1-weighted scans, linearly registered to MNI space to allow for reference to MNI *Z*-axis labels. Image left = anatomical left. (For interpretation of the references to colour in this figure legend, the reader is referred to the web version of this article.)

Compared to control participants, the VPT group displayed increased brain activation in a large cluster with a peak in the right cerebellum, that extended into right lingual, parahippocampal and middle temporal gyri (Fig. 1 and Table 2).

### 3.4 Structural brain differences

VPT participants compared to controls showed decreased grey matter in the left middle temporal gyrus and right superior frontal gyrus/premotor cortex (*p* < 0.05, corrected for multiple comparisons). Inspection of the uncorrected *p* < 0.001 threshold revealed a bilateral pattern of reduced grey matter volume in the MTG and SFG (see Fig. 1 and Table 3).

**Table 3:** Areas of greater grey matter volume in control participants compared to VPT participants.

Controls > VPT	Region	MNI co-ordinates (peak)			*Z*[SPM]
		*x*	*y*	*z*	
FWE corrected (*p* < 0.05)	Left middle temporal gyrus	−68	−4	−15	4.58
	Right premotor cortex	28	0	57	4.57
Uncorrected (*p* < 0.001 voxel level)	Left premotor cortex	−18	−7	45	4.44
	Right middle temporal gyrus	50	−18	−20	4.19

There were no significant differences between the groups in intracranial volume (*t* = 0.66; *p* = 0.514) or total grey matter volume (*t* = 0.744; *p* = 0.462).

### 3.5 Association between structural and functional MRI results

To examine the association between structural and functional neuronal correlates, a series of linear regression analyses were conducted using the SSQ extracted from the cluster of between-group differences in functional brain activation as the dependant variable. Regression analysis was run on all subjects together, in order to allow us to study a greater range of functional activation values. Grey matter volumes (eigenvalues) extracted from all regions of significant between-group grey matter differences (after FWE correction) were used as predictors. Grey matter volume in the right premotor cortex (VPT < Controls) was negatively correlated with BOLD signal and accounted for 33.1% of the variance in activation in the cerebellum (Beta = −0.575; *p* = <0.001). Grey matter volume in the left MTG (VPT < Controls) accounted for 19.4% of the variance in BOLD signal in the same cerebellar cluster (Beta = −0.441; *p* = 0.009). Results of a multiple linear regression revealed that the grey matter volume in left MTG accounted for no additional variance (in addition to that explained by the right premotor cluster) in cerebellar functional activation, and it was removed from the model. As there are strong functional connections between the left premotor cortex and the right-sided cerebellum (Ramnani et al., 2006), we also ran a linear regression analysis using the left sided premotor cluster from the structural analysis as the independent variable and the cerebellar activation as the dependent variable. Grey matter volume in the left premotor area accounted for 14% of the variance in cerebellar activation (Beta = −0.375; *p* = 0.029), although when entered into a multiple regression model with the right premotor cortex it became apparent that the left premotor grey matter volume explained no additional variance in cerebellar BOLD signal in addition to that explained by the right premotor area.

In order to show the functional and structural results in the same image, we created a transformation between the Talairach template and the Dartel template using FSL's FLIRT (Jenkinson et al., 2002). This transformation was then used to map the functional results to the same space as the structural results (Fig. 1).

### 3.6 Associations with IQ

No statistically significant associations were observed between Full Scale IQ and number of non-responses (Spearmans Rho = 0.22; *p* = 0.12), mean response times (Pearsons *r* = 0.29; *p* = 0.11) or intra-individual co-efficient of response time (Pearsons *r* = −0.01; *p* = 0.6) on the motor initiation task. In addition, no associations were observed between Full Scale IQ and SSQ extracted from the cluster of differential brain activation between groups (Pearsons *r* = 0.01; *p* = 0.994).

### 3.7 Correlations with gestational Age

Gestational Age was not correlated with either BOLD activation in the cerebellar cluster (Pearsons correlation: −0.158; *p* = 0.518) or grey matter eigenvalues in the right MTG (−0.003; *p* = 0.991) or right precentral gyrus (−0.099; *p* = 0.688).

## 4 Discussion

Both VPT participants and controls displayed a within-group pattern of brain activation during motor planning, initiation and execution which is consistent with the activation observed in previous studies with different subject samples (Broome et al., 2010). Between-group analyses revealed increased BOLD signal in VPT participants compared to controls in a cluster with its peak in the right cerebellum, that extended into lingual, parahippocampal and middle temporal gyri. No behavioural differences were observed between the two groups during on-line completion of the motor task, and most participants performed at ceiling levels. However, the presence of increased brain activation in the right-sided cerebellum and nearby areas of the temporal lobe suggests that VPT participants recruited additional neural resources in order to complete the task in contrast to term-born peers. We did not see functional activation differences in the periventricular nodes of the motor network as we hypothesised. This may be due to our selection of a sample of VPT participants without grade 3/4 neonatal periventricular haemorrhage, who are at highest risk for structural and functional alterations in the periventricular subcortical structures (Bolisetty et al., 2014, Nosarti et al., 2008). Due to the large voxel size we may have also had less sensitivity to detect activation differences in these smaller grey matter structures. These data suggest possible functional neuroanatomical compensatory processes in cerebellar and cortical areas in a VPT cohort without severe periventricular damage, which also presents several structural brain alterations.

The cerebellum has long been known to have a role in motor control, and research over the last 25–30 years has increasingly pointed towards a role in cognitive function (Middleton and Strick, 2000). Greater activation is seen in regions of the cerebellum that are connected to motor and premotor cortex in controls performing random movement tasks as the complexity of the required movement is increased (Stefanescu et al., 2013). Although participants performed this easy task well, motor and cognitive difficulties are commonly reported in individuals born VPT (Hall et al., 1995, Huh et al., 1998) and thus increased cerebellar activation seen here in the VPT group may reflect greater difficulty in performing the task.

Although in the sample investigated we did not observe significant cerebellar volume differences between the groups, previous studies in larger cohorts found reduced cerebellar volume and growth trajectory in preterm-born adolescents and young adults, which was related to neuropsychological and psychiatric outcome (Allin et al., 2001, Allin et al., 2005, Parker et al., 2008). We did however find a reduction in grey matter volume in right premotor and left middle temporal cortex in the VPT group. Grey matter volume in the premotor cortex explained one third (33.1%) of the variance in activation in the cerebellar cluster. Indeed, inspection of the structural results at a more liberal threshold revealed a bilateral pattern of grey matter volume reductions centred on the premotor cortex, and middle temporal gyrus. The premotor cortex and the cerebellum are critical hubs in motor learning and control networks (Penhune and Steele, 2012, Shadmehr and Krakauer, 2008). The left premotor and primary motor cortices have strong connections with the contralateral cerebellum (Middleton and Strick, 2000). Although we found that a strong negative correlation between grey matter volume in left premotor cortex and right cerebellar activation, right premotor cortical volume explained a greater percentage of the variation in functional BOLD response (in right cerebellum). This implies that the increased activation seen here may be interpreted in the context of increased structural constraints put on bilateral functional motor networks in the VPT group (Salvan et al., 2013). Although it is tempting to infer that cerebellar activation increases are due to premotor cortical deficits, it is also possible that cortical deficits could, in fact, be secondary to cerebellar injury (Limperopoulos et al., 2014).

Increased activation was not seen just in the cerebellum but also in adjacent temporal lobe areas that have been described as being reduced in volume in VPT individuals (Nosarti et al., 2008).

Our results are consistent with the theory that brain regions are capable of performing many cognitive functions, but are constrained by the limited capacity of computational resources (Just and Varma, 2007). It is hypothesised that when a task is sufficiently difficult, exceeding the resources of a particular region, additional brain areas will be recruited to ‘absorb the excess workload’ (Just and Varma, 2007). In the case of altered development in a particular brain structure, such as the dorsal premotor cortex and middle temporal gyrus in the current study, the available resources in these regions are reduced, and so additional regions will be permanently recruited, because ‘the mechanism for shifting a function to a less specialised area is already in place’ (Just and Varma, 2007). This account could explain the increased involvement of the lingual, parahippocampal and middle temporal gyri during performance of the current fMRI paradigm. Alternatively, an increased us of temporal lobe structures in order to facilitate movement ideation may explain the increase in temporal activation seen here (Caffarra et al., 2010). Increased activation observed in the parahippocampal gyri/hippocampus is also consistent with previous data from VPT adults (Lawrence et al., 2010, Salvan et al., 2013).

This study is limited by the small sample size, and relative ease of the task with 71.2% of participants displaying a ceiling effect. These factors may account for the lack of between-group differences in performance. In addition, as the study had missing IQ data for five control participants and only marginal non-significant group differences in IQ between the groups, we cannot exclude the possibility of type II error (i.e., significant difference in IQ between preterm young adults and controls). However, Full Scale IQ was not found to be associated with brain activation in key regions or behavioural response, therefore in this instance we hypothesise it is unlikely that differences in BOLD signal between the groups may reflect potential differences in IQ. On the other hand, if VPT participants in the current study are comparable to controls in terms of IQ, and showed no performance deficits on this task, they may be more accurately described as high-functioning VPT adults. Whether or not the processes of neural plasticity lead to such a positive outcome with more compromised VPT adults is yet to be ascertained. It would have been desirable to have a more even gender distribution across the groups, as this came close to being significantly different between groups. Another limitation of this study is that study participants did not have a neurological examination at assessment; therefore, we were unable to investigate fMRI data in relation to current neurological status.

This study is the first to report the neural correlates of motor planning, initiation and execution following preterm birth in adulthood and showed that despite comparable task performance, VPT young adults displayed increased neural activation in the cerebellum and adjacent temporal lobe areas, which was partly mediated by grey matter volume decreases in premotor cortex. These findings illustrate the remarkable resilience of the preterm brain, suggesting a re-organisation of neural networks to allow successful task performance in VPT adults. Understanding the adult outcome of VPT birth could aid the development of cognitive and neuroprotective remediation strategies to be implemented in younger VPT cohorts.

## Conflicts of interest

The authors declare no conflicts of interest.

## Figures and Tables

**Fig. S1:**
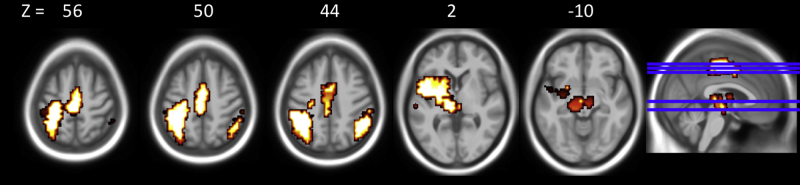
Areas of within group brain activation for Control participants. Areas of within control group activation thresholded at voxel-level *p* < 0.05, cluster-level *p* < 0.01. Left side of images represents the left hemisphere of the brain.

**Fig. S2:**
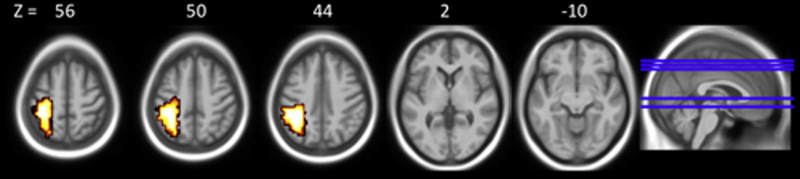
Areas of within group brain activation for VPT participants. Areas of within VPT group activation thresholded at voxel-level *p* < 0.05, cluster-level *p* < 0.01. Left side of images represents the left hemisphere of the brain.

## Footnotes

2 Due to the very narrow age range of VPT participants, there was heterogeneity of variance between groups and Levene's test was significant.

3 There were five instances of missing data in the control group.
